# Exploring Wild *Hordeum spontaneum* and *Hordeum marinum* Accessions as Genetic Resources for Fungal Resistance

**DOI:** 10.3390/plants12183258

**Published:** 2023-09-13

**Authors:** Jaroslava Ovesna, Jana Chrpova, Lucia Kolarikova, Pavel Svoboda, Alena Hanzalova, Jana Palicova, Vojtech Holubec

**Affiliations:** Crop Research Institute, 161 06 Prague, Czech Republic; lucia.kolarikova@vurv.cz (L.K.); pavel.svoboda@vurv.cz (P.S.); hanzalova@vurv.cz (A.H.);

**Keywords:** wild barley, genetic resources, resistance breeding, rust, FHB, net blotch

## Abstract

Crop Wild Relatives (CWRs), as potential sources of new genetic variants, are being extensively studied to identify genotypes that will be able to confer resistance to biotic stresses. In this study, a collection of barley wild relatives was assessed in the field, and their phenotypic variability was evaluated using a Barley Description List, reflecting the identified ecosites. Overall, the CWRs showed significant field resistance to various fungal diseases. To further investigate their resistance, greenhouse tests were performed, revealing that several CWRs exhibited resistance against *Fusarium culmorum*, *Pyrenophora teres*, and *Puccinia hordei* G.H. Otth. Additionally, to characterize the genetic diversity within the collection, DNA polymorphisms at 21 loci were examined. We successfully employed barley-specific SSR markers, confirming their suitability for identifying *H. spontaneum* and even *H. marinum*, i.e., perennial species. The SSR markers efficiently clustered the investigated collection according to species and ecotypes, similarly to the phenotypic assessment. Moreover, SSR markers associated with disease resistance revealed different alleles in comparison to those found in resistant barley cultivars. Overall, our findings highlight that this evaluated collection of CWRs represents a valuable reservoir of genetic variability and resistance genes that can be effectively utilized in breeding programs.

## 1. Introduction

Crop Wild Relative (CWR) species are the ancestors of modern crops. Thus, CWRs constitute unique genetic resources that are suitable for breeding, and they serve as a source of unique genes. During the crop’s domestication and selection, genetic diversity has been reduced, and this reduction has been gradually aggravated by the demand for high crop productivity and crop uniformity, in both the field and the marketplace. CWRs contain some of the necessary genetic diversity required for crop improvement, with the potential to alleviate the genetic bottleneck that is associated with cultivated crops, being an unintended consequence of recurrent selection [1]. As a result, of the breeding process, potentially valuable genetic variants and associated phenotypes have been filtered out of crop gene pools [2]. CWRs possess many desirable traits, especially those that promote resistance to pests and diseases, and tolerance to abiotic stresses, such as drought, heat, and flooding [3]. Such traits, along with nutritional quality and husbandry requirements, can be bred into high-yielding varieties to meet changing environmental and consumer demands [4,5]. The CWRs that are crossable with crop species and that have profitable genes in their genomes represent the most valuable materials for practical breeding.

Among the cereal crops, barley (*H. vulgare* L.) plays an important role as a staple food, as animal feed, and as a basic constituent of beverages. According to FAO data, this ancient grain crop is now grown over a wider range of environments than any other cereal, and its average harvested area is estimated to be 48.9 mil. ha worldwide [6]. The domestication of wild barley probably occurred around 10,000 years ago in the Israel and Jordan region of the Fertile Crescent [7]. Research using haplotype frequency in different geographical parts has led to the inference that there were at least two domestication events, that gave rise to the cultivars grown in Europe and America, while the latter gave rise to the diversity of cultivated crops in Central Asia and the Far East [8]. Wild and domesticated barley differ in several phenotypic characteristics, collectively referred to as the domestication syndrome [9].

Centuries of selection and a period of deliberate breeding have narrowed the genetic basis of this species. The genus *Hordeum*, which includes barley, contains more than 35 species, but out of these, the only direct ancestor of cultivated barley is *Hordeum spontaneum*.

Wild barley has been shown to be a donor of new alleles, conferring resistance to abiotic stresses such as salinity and drought, and biotic stresses such as powdery mildew, which are unique assets for barley improvement, especially in a changing climate [10,11,12].

Wild barley, *Hordeum spontaneum*, a winter annual and a predominantly selfing grass, is native to North Africa, the Middle East, parts of the Indian subcontinent, and Southwest China [13].

Wild barley is found in a wide range of locations, varying from deserts to Mediterranean- and highland-type habitats. The taxon constitutes an important annual element of open herbaceous and park-like vegetation in the Fertile Crescent. Outside of this region, it is mainly restricted to artificial (secondary) habitats and only in widely scattered populations, particularly to the east of its distribution area [14]. In Israel, despite being mainly found in steppe-like formations and park forests, wild barley penetrates into desert (<200 mm annual rainfall) and mountain environments (up to 1600 m altitude), where it has stable populations [15], which attests to the plasticity of the genus. Wild barley is exposed to many environmental stresses, including high temperatures, drought, and high soil salinity, and there are local adaptations to the microclimates in which it grows. Thus, this wild plant is a potential genetic resource for the breeding of stress-tolerant varieties of the cultivated crop [16].

*H. marinum* includes the diploid subspecies *H. m.* ssp. *marinum*, and both the diploid and tetraploid forms of *H. m.* subsp. *gussoneanum*. The tetraploid forms of subsp. *gussoneanum* appear to come from a cross between a diploid *gussoneanum* progenitor and a second related—but unidentified—diploid ancestor. The results reveal the genome structure of the different *H. marinum* taxa and demonstrate the allopolyploid origin of the tetraploid forms of *gussoneanum* [17,18]. *H. marinum* possesses a much higher tolerance to salinity and drought stress compared to cultivated barley. Huang et al. [19] showed that the *H. marinum* genotype used less energy-consuming strategies compared to the barley controls.

Several authors have reported the use of *H. marinum* in breeding programs [20]. A practical application of the present findings is the detection of individual *H. marinum* chromosomes in breeding lines—for example, the material obtained in breeding programs after the hybridization of wheat with this interesting and complex salt- and waterlogging-tolerant group [21,22]. Several substitution lines with improved tolerance have been developed and are available for further use [20,23].

*H. spontaneum*, has been shown to be suitable for improving cultivated barley [24,25] via either conventional or biotech breeding approaches [26]. In the last decade, several new approaches [27] have been introduced to facilitate the broadening of diversity, and to speed up the breeding process. The knowledge gained through genome-wide sequencing and transcriptome analysis [28,29], along with the possibility of introducing new gene variants through traditional approaches, assisted selection, and genome editing [30], is paving the way for more resilient varieties.

Regardless of their importance, CWRs face threats such as intensive agriculture, urban development, pollution, and biological invasions [31,32], and thus they require conservation attention. However, not many genebanks keep them ex situ. Their frequent occurrence under unfavorable conditions and their frequent resistance to common cereal diseases make them perfect potential donors of important genes for cereal breeding.

The aim of this study was to provide a comprehensive evaluation of a unique CWR collection, which included wild spontaneous barley and sea barley (*H. spontaneum* and *H. marinum*), as a possible source of new gene variants. To address this, we performed field and greenhouse resistance evaluations and molecular characterization to assess the CWRs’ variability and their potential value for breeding.

## 2. Results

**Figure 1:**
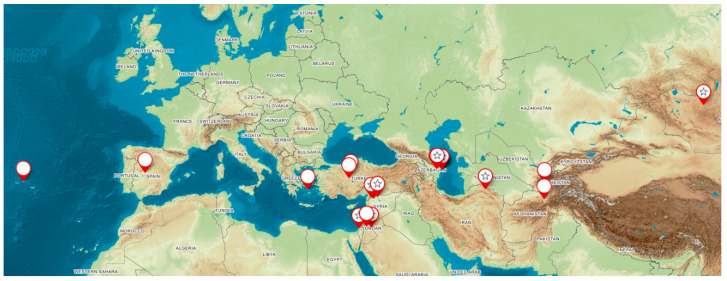
Map of *Hordeum spontaneum* (stars) and *H. marinum* (empty pointers) accessions used for the investigation.

The distribution of the available *Hordeum spontaneum* accessions covers the belt between latitudes 31° and 48° N, from Central Anatolia to Central Asia (Figure 1).

### 2.1. Morphological Phenotyping of the Collection

The phenotyping of 27 morphological characteristics revealed a considerable degree of diversity among the accessions, as is quite often the case in wild species from diverse ecological conditions (Supplementary Table S1). The plant height varied within species and, not surprisingly, between species. The mean plant height varied between 75 and 121 cm within *H. spontaneum*, and between 23 cm and 84 cm within *H. marinum*. Both the height extremes of *H. spontaneum* came from the steppe regions of Azerbaijan: one from the Baku region, and the other from the nearby Shemacha region. The taller genotypes of *H. marinum* belonged to var. *gussoneanum* from Turkey, one from a dry steppe and the other from a saline-disturbed steppe with more rainfall. In general, the phenology of *H. spontaneum* was quite diverse, with lines/accessions completing their heading, flowering, and maturity within a period of 17, 18, and 31 days, respectively, in 2020, and within 7, 13, and 35 days in 2021. The phenology of *H. marinum* was also diverse, with accessions completing their heading, flowering, and maturity within periods of 19, 15, and 28 days, respectively, in 2020, and within 16, 15, and 19 days in 2021. Among the morphological characteristics, there was a remarkable difference in flag leaf position, giving the plants a very different appearance. Another notable difference was the presence and size of the auricles, which could be prominent or almost absent. A high degree of diversity was found in the spike and awn lengths, with both differing among years. Similarly, there was high diversity in the spikelet number and spike density.

**Figure 2:**
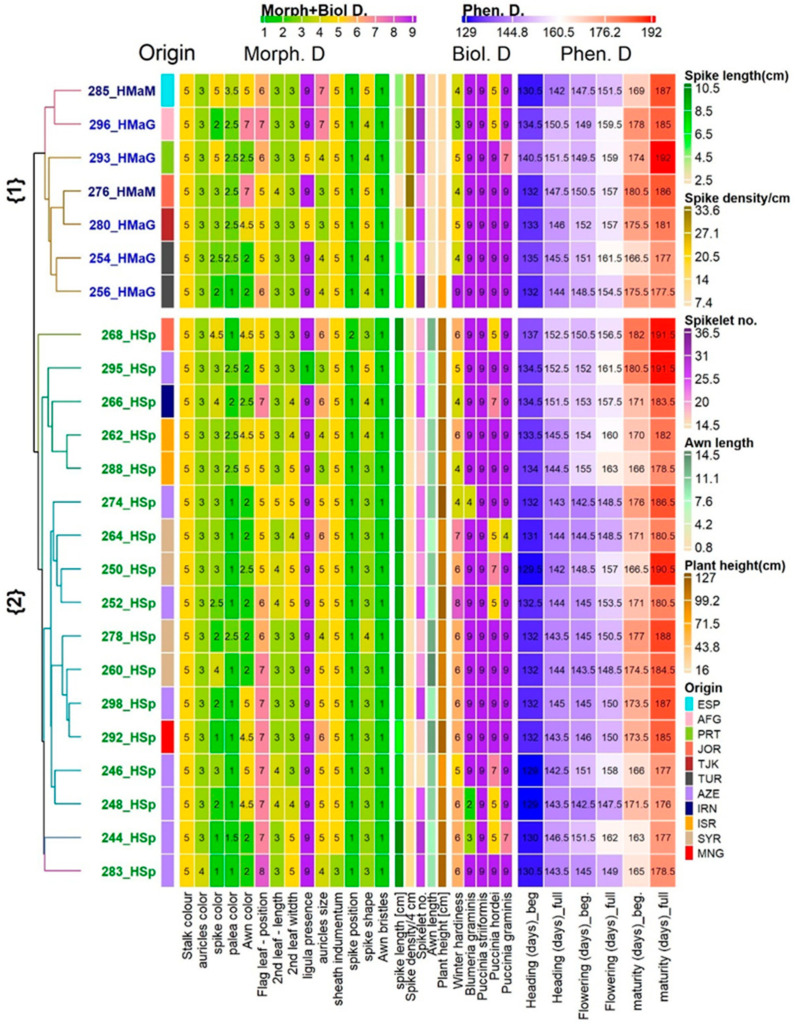
Association of 24 *Hordeum* samples based on a Gower distance dissimilarity matrix and average linkage clustering, along with the values of observed morphological, biological, and phenotypic descriptors. The two groups within the plot denoted by {1} and {2} represent the two main clusters in the dendrogram. The color keys on the left and top sides of the plot indicate the color scale and corresponding values for the selected characteristics. HSp in the row labels denotes the *H. spontaneum* samples, while the HMaM and HMaG labels highlight the *H. marinum* ssp. *marinum* and ssp. *gussoneanum* samples, respectively. Origin stands for abbreviation for country of origin of respective accession: AFG—Afghanistan; AZE—Azerbaijan; ESP—Spain; IRN—Iran; ISR—Israel; JOR—Jordan; MNG—Mongolia; PRT—Portugal; SYR—Syria; TJK—Tajikistan; TUR—Turkey.

**Figure 3:**
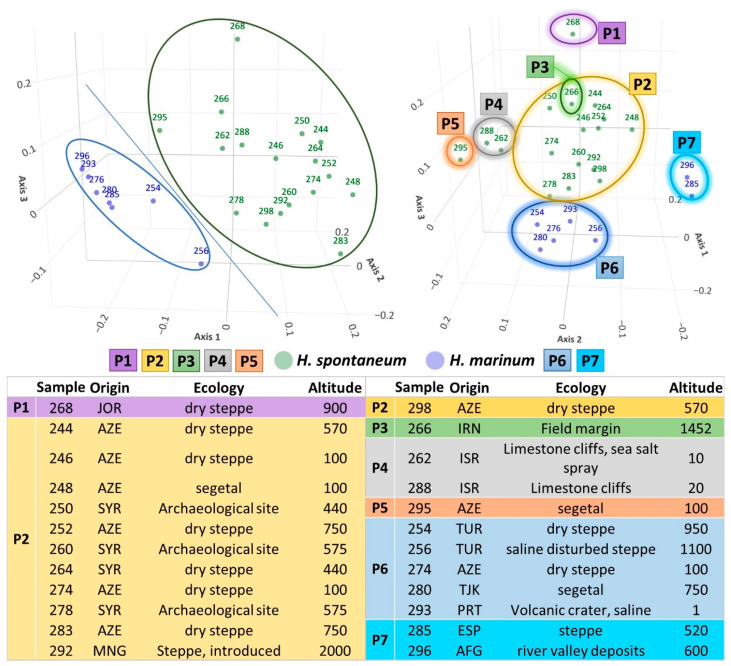
Principal coordinate analysis based on a Gower dissimilarity matrix of phenological estimates for 24 accessions of *H. spontaneum* and *H. marinum*. The plot reveals the relationship between *H. spontaneum* accessions (represented in groups P1–P5) and *H. marinum* accessions (in groups P6–P7), and the diversity within each species. Origin stands for abbreviation for country of origin of respective accession: AFG—Afghanistan; AZE—Azerbaijan; ESP—Spain; IRN—Iran; ISR—Israel; JOR—Jordan; MNG—Mongolia; PRT—Portugal; SYR—Syria; TJK—Tajikistan; TUR—Turkey.

The collected data were utilized to group the accessions based on their similarity, as demonstrated in Figure 2 and Figure 3. The resulting dendrogram classified the genotypes into two main clusters: one was composed solely of *H. spontaneum*, and the other was composed of *H. marinum* accessions (Figure 2). Additionally, the PCoA divided the accessions into seven distinct groups (Figure 3, P1–P7). The first axis and, partially, the third axis were instrumental in separating the *H. spontaneum* and *H. marinum* accessions, with a combined explained variability of 29.91% and 9.48%, respectively, due to the unique features of each species. The second axis (11.335% of the variability) further separated the samples. Despite this, the taxonomic classification of *H. marinum* into the nominated ssp. *marinum* and the derived ssp. *gussoneanum* did not display clear differentiation based on the phenotyping characteristics used in the analysis.

The second and third axes further separated the samples of *H. spontaneum* and *H. marinum*. *H. marinum* samples formed two subgroups, one located in the central part of the plot (Figure 3 P6), comprising *H. marinum* accessions from Turkey, Azerbaijan, Tajikistan, and Portugal. The second subgroup (Figure 3 P7), located on the right side of the plot, comprised accessions from two distant countries (Spain and Afghanistan), but these were collected at similar altitudes. *H. spontaneum* accessions were located in five different clusters (Figure 3 P1–P5), with most of the samples being located in the central part under cluster P2. These samples corresponded to Asian accessions from Azerbaijan, Syria, and Mongolia, with the habitats being the dry steppe, at archaeological sites, and in one case, a segetal environment. Cluster P1 consisted of only one sample, which corresponded to the sole *H. spontaneum* sample of Jordanian origin. Similarly, clusters P3 and P5 each contained a sole sample, and were also distinctly separated from the central cluster. Sample 266 (P3) covered accessions of Iranian provenance, collected at a field margin at high altitude (1452 ASL), while sample 295 (P5) represented the Azerbaijan accession collected at a segetal environment at low altitude (100 ASL). Cluster P4 covered two samples of Israeli provenance collected from limestone cliffs close to sea level.

### 2.2. Evaluation of Resistance against Fungal Pathogens

All the genotypes were evaluated based on field and greenhouse tests.

#### 2.2.1. Field Resistance of the Evaluated Accessions to *Blumeria graminis*, *Puccinia striiformis*, *Puccinia hordei*, and *Puccinia graminis*

Field resistance was monitored during two subsequent years, i.e., 2020 and 2021. No infection by *Blumeria graminis* was recorded in the stands within the 2021 season; however, in the preceding year, three accessions of *H. spontaneum* (01C050928, 01C0509033 and 01C0509031) were severely infected. No symptoms of *Puccinia striiformis* infection were detected across the accessions, indicating a good overall field resistance of both species to the disease. Conversely, *Puccinia hordei* moderately infected half of both the *H. spontaneum* and *H. marinum* species. Seven lines of *H. spontaneum* and three accessions of *H. marinum* were susceptible to *Puccinia hordei*. Only two accession of *H. spontaneum* (01C0509028, 01C0509090) and one of *H. marinum* (01C0509112) were moderately susceptible to *Puccinia graminis*.

Six accessions of *H. marinum* (01C0509067, 01C0509068, 01C0509070, 01C0509107, 01C0509109, 01C0509110) were completely resistant to all four diseases. Eight *H. spontaneum* lines were simultaneously resistant to two of the diseases and only two accessions were fully resistant, and were selected as a possible source of disease-resistance genes (01C0509047, 01C0509089).

#### 2.2.2. Resistance against *Puccinia hordei, Puccinia graminis, Fusarium culmorum* and *Pyrenophora teres* in Greenhouse under Artificial Infection

Resistance to *Puccinia hordei* and *Puccinia graminis* was tested under greenhouse conditions. Only one potential accession that was resistant against *Puccinia graminis* was found under test conditions (*H. spontaneum* 01C0509031). *Puccinia hordei* induced severe symptoms across all the *H. spontaneum* accessions, whereas *H. marinum* accessions were resistant regardless of their origins. The degree of *Fusarium* resistance differed between the *H. spontaneum* genotypes evaluated, ranging from 3 to 7 for accessions originating from the dry steppes of Azerbaijan, according to the visual symptoms score (VSS). These trait values correspond to very susceptible barley cultivars, with a value of up to 7.5 being observed for accessions collected at the archaeological site of Aleppo, Syria, i.e., with resistance levels exceeding the resistances of barley cultivars considered to be sufficiently resistant. In general, six accessions exhibited a good level of resistance and three accessions performed poorly. *H. marinum* genotypes were, on average, moderately resistant to *Fusarium* infection, and only a genotype originally collected in a saline volcanic crater in Portugal was found to be moderately sensitive. No accessions with a rare, high level of resistance were identified. The degree of resistance to different fungal diseases varied. *H. spontaneum* accessions were moderately resistant to *Pyrenophora teres,* with VSSs ranging from 2 to 3, indicating moderate susceptibility. Only one accession was highly susceptible, exhibiting a resistance comparable to that of the sensitive control barley cultivar Adam. *H. marinum* accessions were almost uninfected and could be considered to be resistant in general.

On the basis of the results, we can claim that *H. marinum* accession 01C0509109 showed no disease symptoms under field conditions, and it performed very well in the *Fusarium* and *Pyrenophora teres* resistance tests. Therefore, it can serve as a source of associated genes. Several other accessions may be considered as donors of individual resistance genes.

### 2.3. Evaluation of the Collection Using SSR Markers

A set of 25 *H. spontaneum* (17) and *H. marinum* (8) genotypes, together with control *H. vulgare* cultivars, were screened using 42 SSR markers, 23 of which have previously been described in association with disease resistance loci in barley. The others were found to be useful for diversity studies. They were dispersed across all the barley chromosomes. Within *H. spontaneum,* all of the SSR markers amplified scorable alleles. In *H. marinum*, 34 SSR primers were suitable for loci amplification, which constituted a good number of polymorphic loci within the species. Those markers that were not amplified in *H. marinum* were expected to be associated with resistance loci. In total, exactly 300 different alleles were amplified across the entire set of CWR, with an average allele number of 7.9 per locus. The difference in the number of alleles of different markers was significant. The most polymorphic was the Bmac0125 SSR locus, with 16 alleles. The allele frequencies varied between 2 and 16 (HVRCAGB). Indicators reflecting population diversity, including PIC and H, were also analyzed. Substantial variation was observed among the different loci, of which the highest values of PIC (0.893) were found at the locus BSM40. The highest Hobs (0.773) was calculated for HVM43, and the lowest values for both PIC and Hobs were noted for the HVRCAGB locus (0.083 and 0.141). The Hexp was 0.186, on average. The average PIC was calculated to be 0.245 for *H. marinum*, and 0.685 for *H. spontaneum*. The best discrimination capacity was observed for the markers BMS40 and Bmag0125 (Table 1). With respect to the discrimination capacities of individual multiplexes, multiplex 7 exhibited the capacity to discriminate between all pairs of samples except one, and multiplex 9 discriminated between all of the *Hordeum* samples (Table 1).

**Figure 4:**
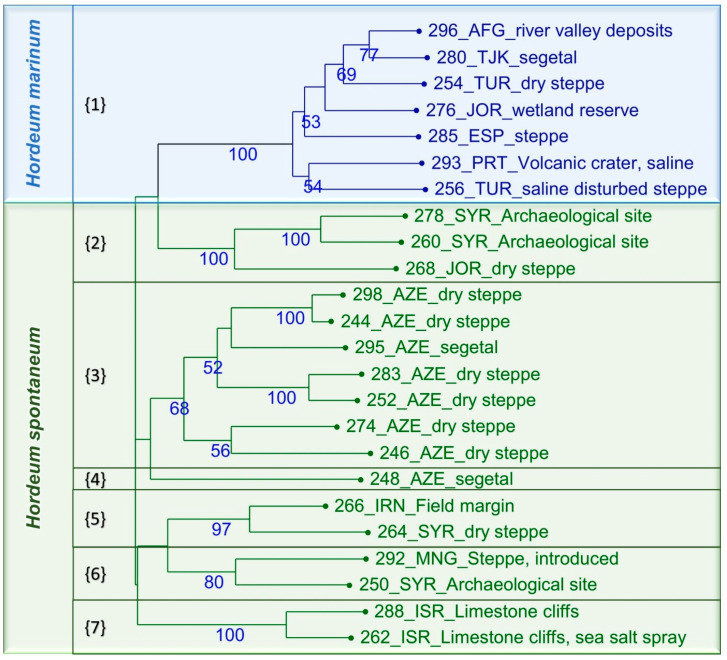
Association between the 24 *Hordeum* accessions, based on a simple matching dissimilarity matrix and unweighted neighbor-joining clustering method. The numbers above each node represent the bootstrap level of confidence (based on 1000 bootstrap replicates). The information at the end of each branch denotes the identification number of the sample, its country of origin, and the habitats of the samples. Numbers enclosed within curly brackets represent 7 main clades. Country of origin of respective accession is stated by its abbreviation: AFG—Afghanistan; AZE—Azerbaijan; ESP—Spain; IRN—Iran; ISR—Israel; JOR—Jordan; MNG—Mongolia; PRT—Portugal; SYR—Syria; TJK—Tajikistan; TUR—Turkey.

To illustrate the relationships between and within species, a cluster analysis was used to generate a dendrogram based on UPGMA (1000 bootstrap replicates). DARwin6 v.6.019 was used to divide the set into seven clades (Figure 4).

**Figure 5:**
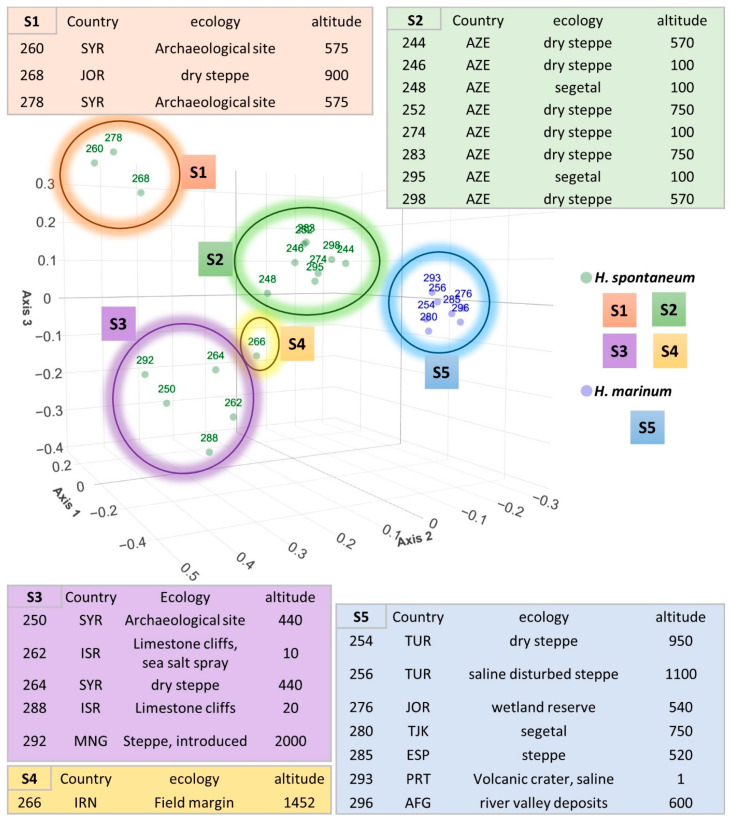
Principal coordinate analysis of 24 *H. spontaneum* and *H. marinum* accessions using SSR markers, based on a simple matching dissimilarity matrix and unweighted neighbor-joining algorithm with 1000 bootstrap replicates. The figure demonstrates the relationship between *H. spontaneum* (in S1–S4) and *H. marinum* (S5) accessions, and the diversity within species. Country of origin of respective accession is stated by abbreviations: AFG—Afghanistan; AZE—Azerbaijan; ESP—Spain; IRN—Iran; ISR—Israel; JOR—Jordan; MNG—Mongolia; PRT—Portugal; SYR—Syria; TJK—Tajikistan; TUR—Turkey.

Clade #1 was composed solely of *H. marinum* accessions. The others consisted of *H. spontaneum* accessions. Clade #2 included accessions collected at archaeological sites in Syria and Jordan, and clades #3 and #4 contained genetic resources collected in Azerbaijan. The last three clades were composed of accessions from dry areas exposed to salt stress and saline spray. The clustering is reliable, as it is supported by bootstrap values of up to 100. A principal coordinate analysis (PCoA) provided a spatial representation of the relative genetic distances between the evaluated genotypes, dividing the accessions in a similar way (Figure 5). The first axis, explaining 28.6%, clearly separated the two species. The second axis, explaining 16.3% of the variability, together with the third axis, reflected the environmental conditions and geographical origins of the CWR. The upper right quadrant (S2) contained the samples from Azerbaijan, while the lower right sector (S4) contained just one sample from Iran. The upper left corner (S1) of the plot comprised three samples from Syria and Jordan, while in the lower left (S3) quadrant, we could find a mixture of samples from Mongolia, Syria, and Israel. Cluster S5, which is separated from the other clusters along the first axis, covers all of the *H. marinum* accessions.

A moderately good measurement fit was observed via the evaluation of the phenological data and SSR. Specifically, the cophenetic correlation between dendrograms based on GS (SSR data) and PS (phenotypic data) was found to be 0.549, and the correlation of the distance matrices calculated through the Mantel test was 0.371.

### 2.4. Evaluation of CWRs Using SSR Markers Associated with Barley Resistance Loci

A subset of SSR and PCR markers previously shown to be associated with the disease resistance loci of cultivated barley was used for the analysis of CWR. The EBmac0679 locus, which was the most polymorphic locus, associated with an FHB resistance locus in barley [33], amplified different alleles compared to both the resistant and susceptible barley cultivars.

Other alleles that were amplified at different loci, identical to those present in resistant cultivars, and amplified by other FHB-linked markers, were only rarely found in the CWR. The same applied for *Pyrenophora teres* and *Puccinia hordei.* The presence of the markers *Rpg1-S, Rpg1-R, Rpg5-S*, and *Rpg5-R*, conferring sensitivity or resistance to *P. graminis*, was verified. However, their presence/absence did not reflect the level of resistance in CRW as the pathogen infection rate was high for all accessions tested. Any of 7 *H. marinum* accession amplified the resistant marker. In case of *H. spontaneum* accessions, 13 out of 17 amplified exactly one of the two (*Rpg1-R* or *Rpg5-R*) fragments (Table 2).

In general, a higher variability of allele sizes was found in the CWRs in comparison to the barley cultivars, indicating that they may be a source of novel DNA sequences and resistance genes.

## 3. Discussion

We aimed to characterize the general diversity of the Hordeum CWR set, which, according to recent studies, could serve as a source of valuable genes needed in current barley breeding [10,34,35]. Special attention was paid to the resistance to biotic stresses, namely resistance to fungal diseases, which is in continuous demand, globally, for all crops [36], since pathogens spreading at a continental scale can have a strong impact on plant health. This is particularly important today, as incursions result in the spread of diseases into new territories in which the disease was previously absent or insignificant [37].

According to previous publications from Korff et al. [38] and Li et al. [39], CWRs were expected to be possible donors of resistance to several diseases, and were therefore evaluated in the field. Field resistance to *Blumeria graminis*, *Puccinia striiformis*, *Puccinia hordei*, and *Puccinia graminis* has been recorded over the years. With one exception, *H. marinum* genotypes were completely resistant against all of the diseases studied under field conditions, as were half of the *H. spontaneum* genotypes. The others were rated as being moderately resistant. Field resistance to fungal diseases in CWRs has previously been reported by several authors [40,41,42]. Therefore, the set of CWRs was further tested for resistance to other fungal diseases in greenhouse tests.

Resistance towards FHB represents a priority trait, and it has been extensively studied. FHB not only causes yield losses, but the pathogen that causes the disease, *Fusarium*, produces mycotoxins that affect human and animal health [43,44]. Unlike *H. marinum*, only three *H. spontaneum* accessions were moderately resistant to FHB, the best of which was No. 01C0509055, originating from the historical site of Aleppo, Syria, which exhibited a slow onset of infection (a long incubation period). The other, coming from a dry steppe, probably had no need to develop mechanisms to allow it to withstand this type of stress. Accession No. 01C0509055 may be considered a potential donor of FHB resistance for breeding activities. Except for one genotype (01C0509112), all of the *H. marinum* accessions were moderately resistant to FHB in the greenhouse tests, probably reflecting their rather humid place of origin, where the pathogen spreads easily and where plants have to cope with pathogen attack, exemplifying their adaptation to environmental factors, as indicated previously [45,46,47]. SSR markers linked to disease resistance in barley did not amplify the same alleles on the studied loci that we found in the resistant barley cultivars Chevron and Frederikson [48]. The size variability of SSRT alleles was considerable. Thus, different alleles probably confer the trait in *H. marinum*. In this case, *H. marinum* may serve as an alternative source of FHB resistance gene(s). As it is known that FHB resistance is a quantitative trait [49], a deeper investigation of the genetic basis of these resources is suggested. Based on this evaluation, *H. marinum* appears to be a suitable source of FHB resistance genes, the exact function and coding sequences of which should be further characterized (Huang et al. [19] or Liu et al. [50]).

*H. spontaneum* has also been reported to be a valuable source of *Puccinia graminis*-resistance genes [51,52]. Nevertheless, only one resistant genotype was found in our collection of *H. spontaneum* (01C0509031) under artificial inoculation conditions, although the same accession exhibited resistance in the field. This may have been caused by different gene sets being expressed in the juvenile and adult stages [53,54]. A low number of rust-resistant *H. spontaneum* L. accessions in other collections was also reported by Sallam et al. [55]. *H. marinum*, on the other hand, was resistant to the disease, as already described in 1996 by Rubiales et al. [40], indicating that the *H. marinum* suppression of appressorium formation had not been investigated to any significant extent until now. We can confirm that *H. marinum* represents a source of genes that confer resistance to *Puccinia hordei*, and that our genotypes can be exploited for this purpose.

As reported previously [56,57], CWRs are a source of genes that confer resistance to *Pyrenophora teres*, another important fungal disease. Although Rehman et al. [41] reported highly resistant *H. spontaneum* accessions, our set of *H. spontaneum* did not exceed the degree of resistance described in some *H. vulgare* genotypes [58]. As *H. spontaneum* coevolved with cultivated barley in the same regions [56], the resistance gene may not vary to a great extent. For the resistant barleys themselves, it has been reported that all chromosomes are occupied by resistance genes [59], and that similar genes and regulatory sequences may occur in wild relatives. The resistance response in barley is multi-level, as it is not determined by a single gene or factor, but rather involves multiple genetic components, with 15 QTLs (= quantitative trait loci) having been described to date [56,60]. The studied *H. marinum* set was generally resistant, and it may also serve as a valuable source of genes that confer resistance towards *P. teres*. This confirms the results of Sato and Takeda [61], who found representatives of the species, especially ssp. *gussoneanum*, to be highly resistant.

The general diversity of the collection was also estimated using SSR markers. The SSR markers currently used for barley genotyping were confirmed to be applicable for *Hordeum spontaneum* investigation, as *H. spontaneum* is a closely related species. Our set of markers was shown to have good discriminatory power. The set of SSR markers enriches those previously shown to be suitable for the genetic characterization of barley-related species described in other studies [62,63,64]. The SSR markers used here were also applicable to *H. marinum*, including the subspecies *gussoneanum*, which might be a diploid or tetraploid perennial species. The only report related to *H. marinum*-specific markers has been described in Jakob et al. [14], who developed chloroplast-specific SSR markers for a phylogeographic study. The transferability of the tested set was found to reach 81%, which is lower than that for *H. spontaneum*, but higher than the transferability of EST markers from wheat to *H. marinum* [65]. The transferabilites of SSR markers from barley to pearl millet were estimated to be 41% and 23.8%, respectively [66]. These numbers are in line with our expectations and reflect the genetic distances between the studied genomes.

On the basis of the calculation of genetic distances employing the length polymorphism of SSR markers, we were able to determine the extent of genetic variation that reflects the local adaptation of CWR. The PCoA SSR clearly distinguishes the material at the species level, mainly because *H. spontaneum* K. Koch and *H. marinum* Hudson differ genomically. While the genome of *H. spontaneum* is closely homologous to *H. vulgare* and is often taxonomically considered to be *H. vulgare* subsp. *spontaneum* (K. Koch) A. et Gr., the genome of *H. marinum* is highly derived. Löve [67], in his genomic concept of *Triticeae*, separated all barleys except *H. vulgare* into the genus Critesion Rafin 1819 (Critesion *marinum* (Hudson) Á.Löve). The separation of the taxa fully corresponds to the groups in the genus Hordeum described by Jacobsen et al. [68].

Within the samples of *H. spontaneum,* the SSR-based PCoA separated a compact cluster of Caucasian accessions from those of Azerbaijan. In addition, within this cluster, it is possible to see a separation between the samples originally collected from the Absheron Peninsula, from the upper part of the cluster characterizing the collections from the eastern inland plains of Shemakha/Shamakhi and Maraza in Azerbaijan. The sites are ecologically different, the former being coastal sands, including urban areas of Baku, and the latter being the deserted steppes of Eastern Transcaucasia.

The smallest cluster contains accessions from the archaeological site of St. Simon in Syria and Jordan. They could have originated from the ragged limestone steppes, or they could have been historically introduced.

The pink cluster combines Near East accessions from diverse localities: archaeological (Aleppo Citadel), roadside (Manbij), and Israel. Special attention should be paid to the collection from Ashkelon, ISR. The sites were very close to each other—about 50 m apart; however, they were ecologically very different. One of the accessions (No. 01C0509046) was collected from limestone pockets in marine cliffs within reach of the salt spray from the sea. The other accession (No. 01C0509047) was collected just above the cliffs in the steppe, on a deep brown soil. Both accessions differed morphologically: the former exhibited a prostrate habit, while the latter, an erect one. In cultivation, they did not differ so much, but the former had a high tendency to lodge, while the latter remained erect. In SSR PCoA, these accessions are clearly separated. The last accession (No. 01C0509053) was collected in the mountain plain of Zagastai, Mongolia. It is clear that it was introduced outside of the *H. spontaneum* distribution area, and it may have appeared in this cluster by chance.

The taxonomic division of *H. marinum* into the nominate ssp. *marinum* and the derived ssp. *gussoneanum* did not show a clear separation [17] based on the phenotyping characteristics used, probably because we only had a limited number of accessions, and both subspecies generally occupy similar habitats within the common, large distribution area. The phenotyping of the morphological characters placed Asian accessions into one central cluster. Accessions from Spain and the Azores were split into separate clusters. It was possible to characterize accession 01C0509068/256 from Pinarbasi, Turkey, as an outlier in both the PCoA and the dendrogram.

PCoA based on SSR markers separated the accessions of sea barley into three closely connected clusters according to their geographical origin: Western Europe (Spain 01C0509111/285 and Azores 01C0509112/293), Turkey and the Near East (01C0509067/254, 01C0509068/256, 01C0509109/276), and Central Asia (Tajikistan 01C0509107/280 and Afghanistan 296). The genetic diversity within the area of distribution seems obvious, with the air distance between the westernmost and easternmost sites being nearly 8000 km, while the Turkish localities are separated from the Central Asian sites by 2800 km. In addition, the Azores site is very isolated, and the genetic divergence could be expected to be even larger. This highlights the importance of nonselective forces in genetic differentiation.

We selected and described new genotypes of CWR that are resistant to fungal diseases. *H. spontaneum* could be used via conventional breeding, as has been shown by other authors [41,69]. Our accessions add to the list of available resources. Conventional breeding approaches may be exploited to transfer the required genes. *H. marinum* has been reported to be crossable with *H. bulbosum*, which could serve as a bridge between *H. vulgare* and *H. marinum* [70]. Although sea barley has mostly been considered as a potential source of genes related to abiotic stresses, here, we report resistance to several fungal diseases. Technological and scientific development has allowed the full sequence analysis of the corresponding genes, and their introduction into related germplasms via genome editing [71]. This reinforces the importance of studying CWRs as a source of genes for crop adaptation to climate change.

Field observations, the measurements of IPGRI descriptors, specialized resistance tests, and molecular markers confirmed the considerable variability of the CWRs evaluated in this study.

## 4. Materials and Methods

### 4.1. Plant Materials

Two subcollections of wild Hordeum deposited in Genebank CRI Prague were selected to investigate variability: 17 lines of 11 accessions of *H. spontaneum*, and 9 accessions of *H. marinum* (Table 3). Passport data for the *Hordeum* accessions are available in the documentation system GRIN Czech (GRIN Czech Release 1.10.3 [13]).

This wild barley collection covers a major part of the natural distribution area of the two species: from the Mediterranean to Middle Asia. Most of the samples originated from the collection activities of the Czech Genebank.

#### Distribution of Species and Habitats

The distribution of the available *Hordeum spontaneum* accessions covers the belt between latitudes 31° and 48° N, from Central Anatolia to Central Asia (Figure 1). Most of the accessions come from the area of the Fertile Crescent or its surroundings. The habitats can be classified as primary (P: natural or near-natural types of steppes, and coastline cliffs) and secondary (S: within fields, settlements, archaeological sites, and introduced). It was possible to characterize the origins of seven accessions as being primary habitats. The accession from Ashkelon was collected separately from two types of very close habitats: a prostrate form from limestone cliffs, in the reach of sea salt spray, and an erect form from the tops of cliffs. The original hypothesis was that there might be genetic differences between these closely connected populations, i.e., potential adaptations in response to the salt spray. Two sites are at altitudes above 1000 m, and the Mongolian site, in particular, is exposed to severe frost and snow-free conditions in winter. In addition, the Caucasian sites (Shemacha and Maraza) are exposed to low winter temperatures (climate maps), and thus they have higher winter hardiness.

The distribution of the available accessions of the sea barley *H. marinum* covers a belt between latitudes 32° and 40° N, from the Azores Islands, over the Mediterranean, to Central Asia. The habitats cover original coastal sites and inland salt marshes, as well as river deposits and dry steppes that are at least temporarily wet. Saline, including alkaline habitats, is promising for this type of abiotic resistance. The sea barley also occupies waste and barren areas within annual and ephemeric vegetation, at low elevation.

### 4.2. Field Evaluation of the CWR Collection

All accessions were evaluated in the field in regeneration row trials using approximately 40–50 plants at the stands of CRI Prague Ruzyně, the Czech Republic. The values of all traits were collected, along with observations of the degree of resistance against locally important fungal phytopathogens according to the Barley Descriptor List [72]. The evaluation of resistance to rusts and powdery mildew was carried out in field trials with natural infection; however, under significant infection pressure from nearby infection fields. The results were summarized over the testing years.

### 4.3. Evaluation of Brown Rust and Stem Rust Resistance in the Greenhouse

Urediniospores of barley brown rust and stem rust leaves were obtained from different cultivars from various trials located across the country and organized by the Central Institute for Supervising and Testing in Agriculture. All isolates of brown rust carried virulence for the resistance genes *Rph1*, *Rph2*, *Rph4*, *Rph6*, *Rph8*, *Rph9* and *Rph12*. Two isolates were virulent to *Rph3*, *Rph5*. *Rph7* was the only gene for which none of the isolates carried virulence.

Isolation of stem rust was performed to differentiate the species of pathogen, a mixture of urediospores from various locality in the Czech Republic was used for the tests.

Rusts from the leaves were inoculated on the susceptible cultivar Adam. When flecks appeared on inoculated leaves, leaf segments with one developing uredinium of each rust sample were transferred to a Petri dish with water and kept in the greenhouse until urediniospores developed. Single-pustule isolates increased on cv. Adam. Seedlings were inoculated with a mixture of isolates in a water suspension of urediniospores. Inoculated plants were kept in the greenhouse in closed glass cylinders to provide an environment of high air humidity for 24 h. Infection types were evaluated according to Stakman et al. [73] 10–14 days after inoculation, during which time the plants were kept in the greenhouse at 18–22 °C. Avirulence was characterized according to infection types 0, 1, and 2, and virulence according to infection types 3 and 4.

### 4.4. Evaluation of Fusarium Head Blight (FBH) Resistance in the Greenhouse

All of the tested samples were planted in the greenhouse in hill plots in three replications. Artificial inoculation of the spikes with the highly pathogenic isolate B of *Fusarium culmorum* [74,75] was performed in the phase of full flowering. The inoculum (conidial suspension 0.8 × 10^7^/mL) was sprayed onto bunches of 10 flowering spikes selected within hill plots on one date. Inoculated spikes were then kept in polythene bags for 24 h. Disease development was supported via irrigation applied to the plots. Head blight symptoms were evaluated on three dates (usually 14, 21, and 28 days after inoculation) using a 1–9 scale, where 9 < 5%, 8 = 5–17%, 7 = 18–30%, 6 = 31–43%, 5 = 44–56%, 4 = 57–69%, 3 = 70–82%, 2 = 83–95%, and 1 > 95% of the spikelets showing FHB symptoms.

### 4.5. Evaluation of Pyrenophora Teres Resistance in the Greenhouse

The responses of seven genotypes of *H. marinum* and nineteen genotypes of *H. spontaneum* to artificial infection with *Pyrenophora teres* was tested in a greenhouse experiment. *H. vulgare* cv. Adam was used as a positive control. Five different monosporic isolates, originating from the Czech Republic, were used for the inoculation. The inoculum (conidial suspension) was prepared using the procedure described by Palicová-Šárová and Hanzalová [76]. The conidial suspension was decanted and adjusted to 3000 spores/mL. Tween 20 (0.08 mL/100 mL) was added to the inoculum before application. The plants were grown in plastic pots at 20 °C. Seedlings were inoculated at the two-leaf stage via spraying with conidial suspension until the suspension ran off. The inoculated seedlings were covered with glass tubes for 24 h to maintain a high humidity. The reaction of the accessions was rated 9–12 days after inoculation using a 1 to 5 rating scale (1–2 = resistant; 3–5 = susceptible).

### 4.6. Genetic Analysis of the CWR Collection

#### 4.6.1. DNA Extraction

DNA from 24 wild barley accessions (Table 2) was extracted from the fresh leaf material using the CTAB method of Saghai-Maroof et al. [77]. Due to their poor germination and consequent inability to be sampled, accessions 01C0509070 and 01C0509110 were excluded from consideration. The integrity of the isolated DNA was assessed using electrophoresis in an agarose gel (0.8%). The purity and concentration of DNA were further determined using an Implen Nanophotometer^®^ P300 (Implen, GmbH, Munich, Germany). The working concentration was set at 100 ng/μL.

#### 4.6.2. SSR Analysis

To perform SSR analysis, 39 microsatellite markers were used under optimized conditions (Table 4). Molecular markers were amplified using multiplex (Table 5) PCR in an automated thermocycler Veriti^TM^ Thermal Cycler (Applied Biosystems, Foster City, CA, USA). The 10 μL reaction mixture contained 2× QIAGEN Multiplex PCR Master Mix (QIAGEN, Hilden, Germany), 5 μM of each primer, and 100 ng of DNA. The amplification profile consisted of the initial denaturation of the template DNA and HotStarTaq DNA polymerase activation at 95 °C for 15 min, followed by 35 cycles of denaturation at 94 °C for 30 s, primer annealing at 55–60 °C (depending on the primer) for 90 s, and product extension at 72 °C for 60 s. The final extension of the amplicons was performed at 60 °C for 30 min. The PCR products were checked by electrophoresis in an agarose gel (2%), and then separated using capillary electrophoresis in an ABI PRISM 3500 Genetic Analyzer (Applied Biosystems, Foster City, CA, USA). The SSR data were processed using GeneMapper software v6 (Applied Biosystems, Foster City, CA, USA).

### 4.7. Screening for Length Variability at Rpg1- and Rpg5-Associated Loci

Twenty-four DNA samples were screened for the presence of two *Rpg* genes (*Rpg1* and *Rpg5*). The associated molecular markers (Table 6) were amplified in an automated Veriti^TM^ Thermal Cycler (Applied Biosystems, Foster City, CA, USA). The 15 μL reaction mixture contained 10× PCR buffer (Qiagen, Hilden, Germany), 2 mM of MgCl_2_ (Qiagen, Hilden, Germany), 200 μM of each dNTP (Invitrogen, Waltham, MA, USA), 333 nM of each primer, 1 U of *Taq* polymerase (Qiagen, Hilden, Germany), and 100 ng of DNA. The amplification profile consisted of an initial denaturation of the template DNA and *Taq* polymerase activation at 94 °C for 5 min, followed by 35 cycles of denaturation at 94 °C for 30 s, primer annealing for 30 s, and product elongation at 72 °C for 1 min. The final extension of the amplicons was performed at 60 °C for 10 min. PCR products were visualized on a 2.5% agarose gel stained with ethidium bromide, using a GeneRuler^TM^ 100 bp Plus DNA Ladder as a reference for amplicon lengths (Fermentas, Waltham, MA, USA).

### 4.8. Statistical Analysis

The SSR data were processed using GeneMapper software v6 (Applied Biosystems). The probability of nonidentity (heterozygosity H) [83] and the polymorphism information content (PIC) [84] were calculated from Equations (1) and (2), respectively:

(1) $$H=1-\sum_{i=1}^{n}p_{i}^{2}$$

(2) $$\mathrm{PIC}=1-H=1-\sum_{i=1}^{n}p_{i}^{2}-\sum_{i=1}^{n-1}\sum_{j=i+1}^{n}2p_{i}^{2}p_{j}^{2}$$

where n represents the number of alleles at a given locus and p_i_/p_j_ represents the frequency of the ith/jth alleles. The number of different SSR patterns (I) was determined, both for each simplex SSR reaction and for the multiplex reactions. The confusion probability (C_j_), i.e., the probability that two randomly selected samples shared the same SSR allele at the jth locus [85], was calculated using Equation (3):

(3) $$C_{j}=\sum_{i=1}^{I}p_{i}\frac{\left(N_{p_{i}}-1\right)}{N-1}$$

where p_i_ represents the frequency of the ith pattern, N represents the sample size, and I represent the total number of patterns generated by the assay. Using the C_j_ values, the discriminatory power of the jth assay was determined by applying Equation (4) [85].

(4) $$D_{j}=1-C_{j}=1-\sum_{i=1}^{I}p_{i}\frac{\left(N_{p_{i}}-1\right)}{N-1}$$

P, i.e., the effective number of SSR patterns per assay, is described by Equation (5) [86].

(5) $$P=\frac{1}{1-D_{L}}$$

where $D_{L}$ is the limit of *D_j_* as *N* tends toward infinity, calculated according to Equation (6).

(6) $$D_{L}=\lim\left(D_{j}\right)=1-\sum_{i=1}^{I}p_{i}^{2}$$

The theoretical number of nondifferentiated pairs of samples for the jth primer was calculated using Equation (7) [85].

(7) $$x_{j}=\left(N\frac{\left(N-1\right)}{2}\right)C_{j}$$

### 4.9. Cluster and Dissimilarity Analyses

Pair-wise distances between the samples’ SSR profiles based on simple matching coefficients [87] were calculated using DARwin6 v6.0.19 software (https://darwin.cirad.fr/ accessed on 7 September 2023), and the results were then used to construct a dendrogram based on the unweighted neighbor-joining algorithm (1000 bootstrap replicates). A cophenetic correlation [88] was used to measure how well the dendrogram reflected dissimilarities within the original data. A principal coordinate analysis (PCoA) was performed on the basis of the dissimilarity matrices following their transformation via a power factor of 0.78215, which was deemed to be suitable for converting dissimilarities, based on a simple matching coefficient, into Euclidean distances.

### 4.10. Phenotypic Data Analysis

The association of samples based on phenotypic data was also performed using DARwin6 software. First, a dendrogram was constructed using UPGMA clustering based on the Gower distance dissimilarity matrix. The dendrogram was then combined with a heatmap to display both the associations of the respective accessions and the corresponding values of the phenotypic traits. As in the case of the SSR data, the dissimilarity matrix transformed by the power factor of 0.64 to the Euclidean distances was used for PCoA analysis of the phenotypic data.

### 4.11. SSR and Phenotypic Data Comparisons

A Mantel test under the ape [89] library of the R program [90] was performed to obtain the level of correlation between the SSR and the phenotypic data. The cophenetic correlation between the dendrograms, both SSR and phenotype based, was also assessed using “R” and the routines within the extended [91] library.

## 5. Conclusions

Wild relative crop species are collected, maintained and characterized because they are considered a valuable source of alternative genes for the improvement of modern varieties. Our investigation not only described newly collected *Hordeum spontaneum* L. and *Hordeum marinum* L accessions, but differentiated them using both IPGRI descriptors and molecular markers according to the species and ecological origin. Although sea barley has mostly been considered as a source of genes related to abiotic stresses, here, we reported resistance to several fungal diseases. The CRW collection of the Czech genebank thus represents a valuable reservoir of genes that could be used in breeding. Based on SSR marker characterization, the resistance differed from those present in the modern (primary) breeding pool. The detailed future characterization of resistance genes via sequencing will allow us to develop new knowledge, allow us, for example, to edit homologous genes in barley or use genetic engineering to transfer them. Thus, we have demonstrated that the investigation and preservation of newly collected genetic resources is still relevant.

## Figures and Tables

**Table 1:** The ability of SSR markers to discriminate between analyzed samples.

	*An*	*I*	*I/N_s_*	*P*	*P%*	*Pu*	*Pu/N_s_*	*D_j_*	*C_j_*	*D_L_*	*x_j_*
BMS02	5	9	0.375	5.747	0.639	-	-	0.862	0.138	0.826	38
Bmag0135	7	8	0.333	3.788	0.474	-	-	0.768	0.232	0.736	64
BMS90	6	7	0.292	3.65	0.521	-	-	0.757	0.243	0.726	67
BLYRCAB	14	15	0.625	10.309	0.687	-	-	0.942	0.058	0.903	16
HVM62	5	9	0.375	5.988	0.665	-	-	0.87	0.13	0.833	36
Bmac0310	10	15	0.625	9.259	0.617	-	-	0.931	0.069	0.892	19
HVM74	5	6	0.25	3.344	0.557	-	-	0.732	0.268	0.701	74
EBmac0906	7	9	0.375	6.41	0.712	-	-	0.88	0.12	0.844	33
BMS32	11	12	0.5	9.901	0.825	-	-	0.938	0.062	0.899	17
BMS18	4	6	0.25	3.509	0.585	-	-	0.746	0.254	0.715	70
HVM60	9	10	0.417	6.41	0.641	-	-	0.88	0.12	0.844	33
EBmac0874	8	8	0.333	3.891	0.486	-	-	0.775	0.225	0.743	62
Bmag0500	6	7	0.292	3.597	0.514	-	-	0.754	0.246	0.722	68
Bmag0173	7	8	0.333	5.747	0.718	-	-	0.862	0.138	0.826	38
BMS40	13	18	0.75	15.152	0.842	-	-	0.975	0.025	0.934	7
HVM30	5	7	0.292	4.292	0.613	-	-	0.801	0.199	0.767	55
Bmac0181	9	13	0.542	8	0.615	-	-	0.913	0.087	0.875	24
HVM67	5	8	0.333	5.236	0.654	-	-	0.844	0.156	0.809	43
HVM43	7	7	0.292	5.051	0.722	-	-	0.837	0.163	0.802	45
Bmac0067	9	13	0.542	5.747	0.442	-	-	0.862	0.138	0.826	38
Bmag0120	6	9	0.375	5.525	0.614	-	-	0.855	0.145	0.819	40
Bmag0136	3	4	0.167	2.551	0.638	-	-	0.634	0.366	0.608	101
Bmag0496	11	14	0.583	11.111	0.794	-	-	0.949	0.051	0.91	14
HVM40	8	12	0.5	7.576	0.631	-	-	0.906	0.094	0.868	26
Bmag0306	12	11	0.458	5.236	0.476	-	-	0.844	0.156	0.809	43
Bmag0112	9	11	0.458	7.042	0.64	-	-	0.895	0.105	0.858	29
HVCMA	3	5	0.208	3.472	0.694	-	-	0.743	0.257	0.712	71
Bmag0394	6	10	0.417	5.747	0.575	-	-	0.862	0.138	0.826	38
EBmac0679	10	12	0.5	7.576	0.631	-	-	0.906	0.094	0.868	26
HVBKASI	4	8	0.333	5.051	0.631	-	-	0.837	0.163	0.802	45
EBmac0602	11	17	0.708	9.901	0.582	-	-	0.938	0.062	0.899	17
Bmag0125	16	18	0.75	14.493	0.805	-	-	0.971	0.029	0.931	8
HVRCABG	2	3	0.125	1.292	0.431	-	-	0.236	0.764	0.226	211
Bmag0807	6	8	0.333	5.525	0.691	-	-	0.855	0.145	0.819	40
EBmac0415	13	14	0.583	5.747	0.41	-	-	0.862	0.138	0.826	38
HVM54	9	13	0.542	8.197	0.631	-	-	0.917	0.083	0.878	23
EBmac0603	11	12	0.5	5.988	0.499	-	-	0.87	0.13	0.833	36
Bmag0692	8	9	0.375	5.319	0.591	-	-	0.848	0.152	0.812	42
Lrk3SNP_Lrk3S4	2	3	0.125	1.988	0.663	-	-	0.518	0.482	0.497	133
MTP 1	23	22	0.917	19.231	0.874	19.231	0.801	0.989	0.011	0.948	3
MTP 2	20	19	0.792	14.493	0.763	14.493	0.604	0.971	0.029	0.931	8
MTP 3	31	19	0.792	16.949	0.892	16.949	0.706	0.982	0.018	0.941	5
MTP 4	33	22	0.917	20.408	0.928	20.408	0.85	0.993	0.007	0.951	2
MTP 5	26	21	0.875	19.231	0.916	19.231	0.801	0.989	0.011	0.948	3
MTP 6	18	15	0.625	7.812	0.521	7.812	0.326	0.909	0.091	0.872	25
MTP 7	30	23	0.958	22.222	0.966	22.222	0.926	0.996	0.004	0.955	1
MTP 8	16	17	0.708	12.5	0.735	12.5	0.521	0.96	0.04	0.92	11
MTP 9	38	24	1	23.81	0.992	23.81	0.992	1	0	0.958	0
MTP 10	30	22	0.917	20.408	0.928	20.408	0.85	0.993	0.007	0.951	2
MTP 11	19	17	0.708	13.699	0.806	13.699	0.571	0.967	0.033	0.927	9

*An*: number of alleles; *N_s_*: number of samples; *I*: number of distinct patterns; *P*: effective number of patterns per assay unit; *%P*: percentage of effective patterns; *Pu*: average effective number of patterns per multiplex; *D_j_*: discriminatory power; *C_j_*: confusion probability; *D_L_*: limit of *D_j_* as *N*→∞; *x_j_*: number of nondifferentiated pairs of samples.

**Table 2:** Molecular marker results obtained using four primer sets designed to detect the presence or absence of *Rpg1* and *Rpg5*. “+” and “-” indicate positive and negative polymerase chain reactions, respectively. Accession numbers highlighted in yellow correspond to *H. marinum*, while white represents *H. spontaneum* accessions.

Accession Number	Our Number	*Rpg1-R*	*Rpg1-S*	*Rpg5-R*	*Rpg5-S*
01C0509028	244	-	-	+	-
01C0509031	246	-	+	-	-
01C0509033	248	-	-	+	-
01C0509055	250	-	-	-	+
01C0509057	252	-	-	+	-
01C0509067	254	-	+	-	+
01C0509068	256	-	+	-	-
01C0509089	260	+	-	-	+
01C0509047	262	-	-	+	-
01C0509090	264	-	-	-	+
01C0509101	266	-	-	+	+
01C0509106	268	+	-	-	+
01C0509031	274	-	+	+	-
01C0509109	276	-	+	-	+
01C0509089	278	+	-	-	+
01C0509107	280	-	+	-	+
01C0509057	283	-	-	+	-
01C0509111	285	-	+	-	+
01C0509047	288	-	-	+	-
01C0509053	292	-	-	-	-
01C0509112	293	-	+	-	+
01C0509033	295	-	-	+	-
01C0509108	296	-	+	-	-
01C0509028	298	-	-	+	-

**Table 3:** Items in the CWR collection of *Hordeum spontaneum* and *Hordeum marinum* in the Czech Genebank (collected by Vojtěch Holubec, CRI, CZ, except those with a Nordic GB donor number N). Origin stands for abbreviation for country of origin of respective accession: AFG—Afghanistan; AZE—Azerbaijan; ESP—Spain; IRN—Iran; ISR—Israel; JOR—Jordan; MNG—Mongolia; PRT—Portugal; SYR—Syria; TJK—Tajikistan; TUR—Turkey.

Acc. No.	Regen. No	Taxon	Origin	Locality	Latitude	Longitude	Altitude	Ecology	Habitat Type: Primary/Secondary	Coll. No./Donor No.
01C0509028	244	*H. spontaneum*	AZE	Shemacha-Maraza	40°33′37.633″ N	48°45′45.465″ E	570	dry steppe	P	E200
01C0509028	298	*H. spontaneum*	AZE	Shemacha-Maraza	40°33′37.633″ N	48°45′45.465″ E	570	dry steppe	P	E200
01C0509031	246	*H. spontaneum*	AZE	Baku-Chanlar	40°21′48.870″ N	49°47′55.906″ E	100	dry steppe	S	E215
01C0509031	274	*H. spontaneum*	AZE	Baku-Chanlar	40°21′48.870″ N	49°47′55.906″ E	100	dry steppe	S	E215
01C0509033	248	*H. spontaneum*	AZE	Baku, city	40°25′12.789″ N	49°49′18.252″ E	100	segetal	S	E216
01C0509033	295	*H. spontaneum*	AZE	Baku, city	40°25′12.789″ N	49°49′18.252″ E	100	segetal	S	E216
01C0509046	262	*H. spontaneum*	ISR	Ashkelon, prostrate	31°39′48.799″ N	34°32′42.246″ E	10	Limestone cliffs, sea salt spray	P	E509
01C0509047	288	*H. spontaneum*	ISR	Ashkelon, erect	31°39′48.799″ N	34°32′42.246″ E	20	Limestone cliffs	P	E512
01C0509053	292	*H. spontaneum*	MNG	Zagastai, Zavchan	48°48′0.000″ N	97°58′0.000″ E	2000	Steppe, introduced	S	E786
01C0509055	250	*H. spontaneum*	SYR	Aleppo, Citadel	36°11′57.108″ N	37°9′43.965″ E	440	Archaeological site	S	E926
01C0509057	252	*H. spontaneum*	AZE	Maraza	40°30′33.780″ N	48°57′1.615″ E	750	dry steppe	P	JAN2/35
01C0509057	283	*H. spontaneum*	AZE	Maraza	40°30′33.780″ N	48°57′1.615″ E	750	dry steppe	P	JAN2/35
01C0509089	260	*H. spontaneum*	SYR	St.Simeon	36°20′0.850″ N	36°50′37.439″ E	575	Archaeological site	S	E1292
01C0509089	278	*H. spontaneum*	SYR	St.Simeon	36°20′0.850″ N	36°50′37.439″ E	575	Archaeological site	S	E1292
01C0509090	264	*H. spontaneum*	SYR	Manbij	36°30′35.466″ N	37°57′37.566″ E	440	dry steppe	P	E1342
01C0509101	266	*H. spontaneum*	IRN	Chukanlu	37°35′24.200″ N	57°51′36.500″ E	1452	Field margin	S	18/56
01C0509106	268	*H. spontaneum*	JOR	Amman, E of Belem	31°57′5.650″ N	35°55′26.265″ E	900	dry steppe	S	
01C0509067	254	*H. marinum* ssp. *gussoneanum*	TUR	Tolkoy, Ankara Prov.	39°38′12.501″ N	33°3′9.550″ E	950	dry steppe	P	E937
01C0509068	256	*H. marinum* ssp. *gussoneanum*	TUR	Pinarbasi SW, Kayseri Prov.	39°12′27.762″ N,	32°43′12.546″ E	1100	saline disturbed steppe	P	E955
01C0509070	258	*H. marinum* ssp. *gussoneanum*	TUR	Tutak, Nehri R., Agri Prov.	39°32′21.681″ N	42°46′21.900″ E	1550	saline disturbed steppe	P	E971
01C0509107	280	*H. marinum* ssp. *gussoneanum*	TAI	Sharora, W Dushambe	38°30′29″ N	68°42′29″ E	750	segetal	S	N6507_1
01C0509108	296	*H. marinum* ssp. *gussoneanum*	AFG	Kataghan Prov.	36°7′12.338″ N,	68°40′54.702″ E	600	river valley deposits	P	N6509_2
01C0509109	276	*H. marinum* ssp. *marinum*	JOR	Azraq Oasis	31°50′1.574″ N	36°49′16.644″ E	540	wetland reserve	P	N6820_4
01C0509110	290	*H. marinum* ssp. *marinum*	GRC	Tinos Island	37°32′40.976″ N	25°9′12.919″ E	5	coastland	P	N6824_5
01C0509111	285	*H. marinum* ssp. *marinum*	ESP	Arroyo de Los Fraeles, S of Talavera de La Reina	39°54′29″ N	4°48′29″ W	520	steppe	P	N7295_6
01C0509112	293	*H. marinum* ssp. *gussoneanum*	PRT	Terceira Isl., Acores	38°43′16.298″ N,	27°13′3.490″ W	450	Volcanic crater, saline	P	N90237_7

**Table 4:** Molecular markers used in the SSR assay to detect length polymorphisms of microsatellites associated with resistance to pathogens (Pt = *Pyrenophora teres*; Ph = *Puccinia hordei*; F = *Fusarium*). The color of each marker name shows the fluorochrome with which the respective primer is labelled (green—VIC, red—PET, yellow—NED and blue—6-FAM).

Marker Name	Product Length (bp)	Primer Sequence	Annealing Temperature	Association with Resistance (Pt, Ph, F)	Reference
**BMS32**	186–228	F: 5′-GGA TCA AAG TCC GGC TAG-3′	55 °C	-	[34]
		R: 5′-TGC GGG CCT CAT ACT GAC-3′			
**BMS40**	176–225	F: 5′-AGC CCG ATC AGA TTT ACG-3′	55 °C	-	[34]
		R: 5′-TTC TCC CTT TGG TCC TTG-3′			
**BMS90**	178–225	F: 5′-ACA TCA ACC CTC CTG CTC-3′	60 °C	-	[34]
		R: 5′-CCG CAC ATA GTG GTT ACA TC-3′			
**EBmac0415**	225–285	F: 5′-GAA ACC CAT CAT AGC AGC-3′	60 °C	F	[34]
		R: 5′-AAA CAG CAG CAA GAG GAG-3′			
**EBmac0602**	187–221	F: 5′-GAT TGG AGC TTC GGA TCA C-3′	60 °C	F	[34]
		R: 5′-CCG TCT AGG GAG AGG TTC TC-3′			
**EBmac0603**	134–184	F: 5′-ACC GAA ACT AAA TGA ACT ACT TCG-3′	58 °C	Ph	[34]
		R: 5′-TGC AAA CTG TGC TAT TAA GGG-3′			
**EBmac0679**	95–148	F: 5′-ATT GGA GCG GAT TAG GAT-3′	60 °C	F	[34]
		R: 5′-CCC TAT GTC ATG TAG GAG ATG-3′			
**EBmac0874**	147–205	F: 5′-AAC CAT TCC TCA CCC AGG-3′	55 °C	Pt	[34]
		R: 5′-GTG AAT GAT GTT GAG GAC ATT G-3′			
**EBmac0906**	140–156	F: 5′-CAA ATC AAT CAA GAG GCC-3′	55 °C	Pt	[35]
		R: 5′-TTT GAA GTG AGA CAT TTC CA-3′			
**HVBKASI**	179–201	F: 5′-ATT GGC GTG ACC GAT ATT TAT GTT CA-3′	60 °C	F	[78]
		R: 5′-CAA AAC TGC AGC TAA GCA GGG GAA CA-3′			
**HVCMA**	125–139	F: 5′-GCC TCG GTT TGG ACA TAT AAA G-3′	60 °C	-	[78]
		R: 5′-GTA AAG CAA ATG TTG AGC AAC G-3′			
**HVM30**	118–154	F: 5′-AGT GGG GAA TGA GAG AAT GG-3′	55 °C	-	[79]
		R: 5′-TGC TTG TGG GTC ATC ACA C-3′			
**HVM40**	141–216	F: 5′-CGA TTC CCC TTT TCC CAC-3′	60 °C	-	[79]
		R: 5′-ATT CTC CGC CGT CCA CTC-3′			
**HVM43**	200–235	F: 5′-GGA TTT TCT CAA GAA CAC TT-3′	55 °C	Pt	[35]
		R: 5′-GCG TGA GTG CAT AAC ATT-3′			
**HVM54**	155–178	F: 5′-AAC CCA GTA ACA CCG TCC TG-3′	60 °C	F	[79]
		R: 5′-AGT TCC CTG ACC CGA TGT C-3′			
**HVM60**	99–120	F: 5′-CAA TGA TGC GGT GAA CTT TG-3′	55 °C	-	[79]
		R: 5′-CCT CGG ATC TAT GGG TCC TT-3′			
**HVM62**	231–265	F: 5′-TCG CGA CCA GAC GAG AAG-3′	60 °C	-	[79]
		R: 5′-AGC TAG CCG ACG ACG CAC-3′			
**HVM67**	97–129	F: 5′-GTC GGG CTC CAT TGC TCT-3′	55 °C	-	[79]
		R: 5′-CCG GTA CCC AGT GAC GAC-3′			
**HVM74**	180–196	F: 5′-AGGAAGTCATTGCGTGAG-3′	60 °C	Pt	[79]
		R: 5′-TGATCAAGAATGATAACATGG-3′			
**HVRCABG**	120–124	F: 5′-TTT AAA AGA AAA GTG AAT GGC-3′	60 °C	F	[79]
		R: 5′-TAA TGA AGA ATG AGG AGA AGC-3′			
**Lrk3SNP/** **Lrk3S4**	261–297	F: 5′-ATC CGC AGG ATG CCC-3′	58 °C	Ph	[80]
		R: 5′-TTG GCC CAA TCT CTT GC-3′			
**BMS02**	207–225	F: 5′-AGA GTA GTG GAA AGA AAG TT-3′	60 °C	-	[34]
		R: 5′-TGG TAG TGA GAT GAG GTG AC-3′			
**BLYRCAB**	136–209	F: 5′-ACA CCT TCC CAG GAC AAT CCA TTG-3′	60 °C	-	[34]
		R: 5′-AGC ACG CAG AGC ACC GAA AAA GTC-3′			
**Bmac0067**	149–216	F: 5′-AAC GTA CGA GCT CTT TTT CTA-3′	60 °C	Pt	[35]
		R: 5′-ATG CCA ACT GCT TGT TTA G-3′			
**Bmac0181**	158–186	F: 5′-ATA GAT CAC CAA GTG AAC CAC-3′	55 °C	Pt	[35]
		R: 5′-GGT TAT CAC TGA GGC AAA TAC-3′			
**Bmac0310**	135–188	F: 5′-CTA CCT CTG AGA TAT CAT GCC-3′	60 °C	Pt	[35]
		R: 5′-ATC TAG TGT GTG TTG CTT CCT-3′			
**Bmag0112**	155–203	F: 5′-CCC GTG ATA TAT TAA GAT CAT G-3′	60 °C	Pt	[35]
		R: 5′-AGG GGG AGA TCT TCT CTG-3′			
**Bmag0120**	219–258	F: 5′-ATT TCA TCC CAA AGG AGA C-3′	60 °C	Pt	[35]
		R: 5′-GTC ACA TAG ACA GTT GTC TTC C-3′			
**Bmag0125**	119–152	F: 5′-AAT TAG CGA GAA CAA AAT CAC-3′	60 °C	F	[35]
		R: 5′-AGA TAA CGA TGC ACC ACC-3′			
**Bmag0135**	138–168	F: 5′-ACG AAA GAG TTA CAA CGG ATA-3′	60 °C	-	[35]
		R: 5′-GTT TAC CAC AGA TCT ACA GGT G-3′			
**Bmag0136**	197–201	F: 5′-GTA CGC TTT CAA ACC TGG-3′	60 °C	-	[35]
		R: 5′-GTA GGA GGA AGA ATA AGG AGG-3′			
**Bmag0173**	109–153	F: 5′-CAT TTT TGT TGG TGA CGG-3′	55 °C	Pt	[35]
		R: 5′-ATA ATG GCG GGA GAG ACA-3′			
**Bmag0306**	126–176	F: 5′-ATG TAC AAG TAG CTA TGT GTT TGA-3′	60 °C	Pt	[35]
		R: 5′-CAC ATC AAG ATA ACA AGA GAA GA-3′			
**Bmag0394**	160–182	F: 5′-AAT TCA TCA CAA CAA GAT AGG A-3′	60 °C	-	[35]
		R: 5′-AAT TGA TCT CCC TCT CTC TAT G-3′			
**Bmag0496**	173–229	F: 5′-AGTATAACCAACAGCCGTCTA-3′	60 °C	Pt	[35]
		R: 5′-CTATAGCACGCCTTTGAGA-3′			
**Bmag0500**	142–191	F: 5′-GGG AAC TTG CTA ATG AAG AG-3′	55 °C	-	[35]
		R: 5′-AAT GTA AGG GAG TGT CCA TAG-3′			
**Bmag0692**	158–185	F: 5′-GCA AGG TAT CTC TTG TAT TTT G-3′	58 °C	Ph	[35]
		R: 5′-TGG CAT CTA CAA TCT AAA ACA-3′			
**Bmag0807**	97–113	F: 5′-GGATATAAGGGTCCATAGCA-3′	60 °C	Pt, F	[35]
		R: 5′-AATTACATCAAATAGGCTCCA-3′			
**BMS18**	133–141	F: 5′-GTC CTT TAC GCA TGA ACC GT-3′	55 °C	-	[34]
		R: 5′-ACA TAC GCC AGA CTC GTG TG-3′			

**Table 5:** SSR markers used in multiplexes; the color indicates the fluorescent dye used (blue: FAM; green: VIC; yellow: NED; red: PET).

Multiplex Number	Markers Included	Multiplex Number	Markers Included
MTP 1	**BMS02**	MTP 6	**Bmac0067**
	**Bmag0135**		**Bmag0120**
	**BMS90**		**Bmag0136**
	**BLYRCAB**		
		MTP 7	**Bmag0496**
MTP 2	**HVM62**		**HVM40**
	**Bmac0310**		**Bmag0306**
	**HVM74**		
		MTP 8	**Bmag0112**
MTP 3	**EBmac0906**		**HVCMA**
	**BMS32**		**Bmag0394**
	**BMS18**		
	**HVM60**	MTP 9	**EBmac0679**
			**HVBKASI**
MTP 4	**EBmac0874**		**EBmac0602**
	**Bmag0500**		**Bmag0125**
	**Bmag0173**		
	**BMS40**	MTP 10	**HVRCABG**
			**Bmag0807**
MTP 5	**HVM30**		**EBmac0415**
	**Bmac0181**		**HVM54**
	**HVM67**		
	**HVM43**	MTP 11	**EBmac0603**
			**Lrk3SNP/Lrk3S4**
			**Bmag0692**

**Table 6:** Molecular markers used in the PCR assay to detect amplicons associated with resistance/susceptibility to *Puccinia graminis*.

Marker Name	Product Length (bp)	Primer Sequence	Annealing Temperature	Reference
*Rpg1-R*	669	F: 5′-CGGCTAATCACATCAAGTAA-3′	60 °C	[81]
		R: 5′-AGCCCATCATCAATAGACAA-3′	60 °C	
*Rpg1-S*	487	F: 5′-GGCTAATCACATCAAGGTT-3′	60 °C	[82]
		R: 5′-CCACGACCAATTATGTTCTG-3′	60 °C	
*Rpg5-R*	1046	F: 5′-CTGCTGGCACAGAGTCTGCCTTGAG-3′	60 °C	[81]
		R: 5′-ACTCTCGGGTCTGAAGTTCCGTGTG-3′	60 °C	
*Rpg5-S*	840	F: 5′-CTGCTGGCACAGAGTCTGCCTTGAG-3′	60 °C	[81]
		R: 5′-CCCGAGGTTTGCGATGAAGAGAGTC-3′	60 °C	

## Data Availability

Further information is available from the corresponding authors upon reasonable request.

## Supplementary Materials

The following supporting information can be downloaded at: https://www.mdpi.com/article/10.3390/plants12183258/s1, Table S1: Phenotyping of *Hordeum spontaneum* and *Hordeum marinum* during 20220 and 2022 according to the descriptor list.
